# Point-of-care ultrasound: An emerging clinical tool to enhance physical assessment

**DOI:** 10.1097/01.NPR.0000841944.00536.b2

**Published:** 2022-07-26

**Authors:** Courteney D.M. Fraleigh, Elsie Duff

**Affiliations:** **Courteney Fraleigh** is a Master of Nursing student at the University of Manitoba, Winnipeg, Manitoba, Canada.; **Elsie Duff** is an assistant professor of graduate nursing at the University of Manitoba, Winnipeg, Manitoba, Canada.

**Keywords:** bedside ultrasound, NP, POCUS, point-of-care ultrasound, primary care, rural and remote nursing

## Abstract

Point-of-care ultrasound (PoCUS) is a cost-effective diagnostic technology that, with training, is accessible, portable, and a convenient diagnostic modality to complement physical assessments. PoCUS is beneficial in that it can reduce the number of imaging tests required, while also mitigating barriers to healthcare for rural and remote communities.

**Figure:**
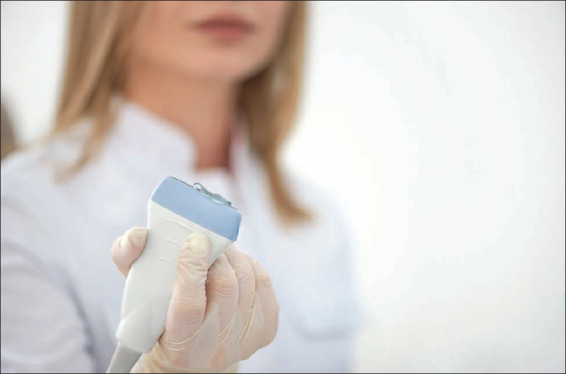
No caption available.

NPs are often confronted with clinical uncertainty that requires further diagnostic evaluation to reason between differential diagnoses. NPs working in primary care must simultaneously assess, diagnose, and treat individuals of all ages who present with acute and/or chronic conditions.^1^ NPs utilize health history and physical assessment as the foundation of patient encounters, then support their assessments with diagnostic technologies. Point-of-care ultrasound (PoCUS) is a technology used to complement patient assessment and diagnostic reasoning that has been utilized in ED settings for more than 3 decades. Portable ultrasound is a reliable bedside technology that can be utilized with accuracy by certified NPs to benefit patient care in both specialty and primary care settings.^2^-^9^ This article reviews the practical bedside applications of PoCUS for certified NPs working in primary care settings as well as its benefits in rural and remote communities.

## PoCUS

PoCUS equipment is portable and used as a diagnostic tool by certified healthcare professionals; it enhances patient assessments within a variety of healthcare settings.^1^,^6^,^10^ PoCUS is conducted at the bedside using a portable ultrasonography machine or handheld device to discern between clinical hypotheses. When conducted by a certified provider, portable ultrasound systems have reliable equivalent accuracy to diagnostic ultrasonography exams completed under the direction of a radiologist within an imaging department.^1^ Thereby, the use of PoCUS has the potential to reduce the number of conventional imaging tests.^1^ PoCUS was initially implemented in critical care approximately 3 decades ago. Its use has been evolving in ambulatory and prehospital practice environments over the last 10 years.^6^,^11^ The uptake of PoCUS among physicians, specialists, and, more recently, paramedics and advanced practice providers (including NPs) has made PoCUS an essential complement to both outpatient and inpatient physical assessments.^1^,^11^

## PoCUS in ambulatory care

PoCUS is used to build upon the results of relevant history and physical assessment to assist the NP in providing a timely, yet accurate, diagnosis.^12^ PoCUS is validated, based on its accuracy and diversity of use, as a tool through which certified practitioners can optimize their clinical judgment and decision-making.^4^,^5^,^12^ Typically, PoCUS is used to efficiently answer a specific clinical question (such as, does my patient have pneumonia?); it is not validated for systemic exam of multiple organs, which requires clinical dictation of results by a radiologist.^9^,^13^

A study in Ireland found that average wait times for certain patients for computed tomography (CT) scans and ultrasound imaging in outpatient settings is 13 weeks, which significantly impacts the assessment accuracy, the potential for rapid decompensation of the patient's condition, and overall healthcare costs.^14^ The uptake of noninvasive PoCUS for routine clinical presentations in primary care has significantly increased over the last 10 years and is expected to continue to expand due to the timely, low-cost, and accurate results offered by this technology when used by certified providers.

Historically, PoCUS was regarded as an essential clinical diagnostic tool in acute care settings, including EDs and ICUs.^1^,^15^ More recently, its implementation was found to be beneficial in other areas, such as prehospital, primary care, and family medicine settings (see *PoCUS benefits and limitations in primary care*).^1^,^10^ PoCUS is being used for clinical presentations to determine its complementary use with a focused health history and physical exam. PoCUS enhances the standard assessment skills of certified primary care practitioners and prompts expedited referrals and accelerated clinical management of recurrent illnesses.

### Rural and remote communities

The implementation goes beyond expedited referrals given that there are over 360,000 practicing NPs within Canada and the US, and up to 50% of this workforce in some provinces or territories is situated in rural and remote communities.^16^,^17^ In Canada, approximately 20% of the population lives in rural and remote communities.^18^ In the US, 85% of NPs specialize in primary care to comprise a significant portion of the interprofessional team in rural and remote communities.^16^ Patients and providers in rural and remote locations have limited access to healthcare and are often confronted with unique challenges.^19^,^20^ Patients in rural communities experience barriers to equitable healthcare, such as travel constraints and incurred costs for services such as conventional diagnostic imaging.^7^,^19^,^20^ Although these barriers are cumbersome for marginalized rural and remote communities, clinical challenges could be reduced by deploying portable and accessible bedside ultrasound devices to be used by certified providers.^7^ It is essential that NPs be trained and educated to utilize this valuable ultrasound skill, particularly given the employment rate of NPs in rural and remote communities, which makes them the fundamental access point for healthcare in these areas.^21^

The use of PoCUS assessment within rural and remote communities could mitigate some of the systemic barriers and decrease the burden for patients who may require additional testing for their presenting condition. Research has shown that PoCUS is beneficial to physicians certified in PoCUS in various clinical settings, but the literature is limited on the implications for NPs and within primary care and rural settings.

Rural nurses and midwives in Indonesia effectively improved patient-care outcomes and addressed barriers to medical care during a physician shortage crisis using PoCUS as a multidisciplinary diagnostic modality.^22^ Other studies involving the use of PoCUS by members of the healthcare team have demonstrated the ease of use, portability, and improved time to diagnosis among outpatients; these factors are fundamental to its practicality and integration into underserved, resource-limited communities.^7^,^20^ Moreover, certified practitioners using PoCUS can improve diagnostic timing without compromising clinical accuracy, while also eliminating the safety issues and costs associated with unnecessary, invasive tests.^1^,^7^,^20^ In the appropriate clinical context, the use of ultrasound eliminates patients' exposure to unnecessary harmful radiation, such as those associated with CT scans. As a primary care clinical tool, PoCUS can be used to complement physical assessments to expedite diagnosis, reduce time to treatment, and precipitate timely referrals by trained healthcare professionals.

## Benefits of PoCUS

PoCUS has many benefits above and beyond diagnostic accuracy, including cost savings to patients, providers, and organizations. The costs of diagnostic imaging, such as CT scans or MRIs, are substantially higher than the overall inclusive costs of PoCUS assessment. In Canada, healthcare expenditures per person in northern remote communities are more than double those in urban centers.^23^ Rising healthcare expenditures are exacerbated by increasing transportation expenses, higher rates of comorbidities, and insufficient access to healthcare services in rural and remote communities.^24^ Given the large composition of NPs serving these communities, it is essential that NPs be certified to implement and utilize cost-saving and modern diagnostic modalities, such as PoCUS. As such, NPs can abate healthcare spending and reduce the burden on patients within these vulnerable populations. NPs can also minimize time spent ordering diagnostics and reduce the number of return visits for diagnostic follow-up appointments by implementing PoCUS into their practice.

## PoCUS education and training

PoCUS education and training is essential and dependent upon regulatory certification requirements within the clinical practitioner's jurisdiction. For example, the American Registry for Diagnostic Medical Sonography in the US and the Canadian Point of Care Ultrasound Society (CPoCUS) in Canada set the PoCUS education standards.^25^ One option for PoCUS certification is introductory and independent practitioner courses offered through the CPoCUS. Together, these courses comprise anatomy review, online lectures, and hands-on experiences with subsequent proctored scans. There are six steps to completing the independent practitioner course, which includes logged clinical scans with the supervision of a proctor with hands-on and written exams. The fee for certification varies by jurisdiction, and workplace depending, may be reimbursed. PoCUS education is fundamental to ensure clinical accuracy and interpretation for patient safety.

## Indications for use in primary care

PoCUS is an essential clinical tool in prehospital and primary care practice, particularly for obstetrics and abdominal assessments.^26^ From the patient perspective, in one study, up to 95% of patients felt that PoCUS improved their primary care health visit, with 45% feeling that the provider-patient relationship had improved.^27^ There were no patients within the study that felt the PoCUS exam as part of the physical assessment provided a negative experience.^27^ Bedside ultrasound can also be used for other indications, such as cardiopulmonary, soft tissue, and musculoskeletal evaluation, in primary care and resource-limited communities (see *Indications for use of ultrasound in primary care*).

### Cardiovascular

Rapid evaluation of acute and chronic cardiac conditions can lead to early management and improved patient outcomes.^4^,^28^ PoCUS can detect cardiac issues such as pericardial effusion and decreased systolic function without minimizing accuracy and safety.^29^ For conditions such as heart failure, PoCUS serves as a sensitive diagnostic modality with improved time to diagnosis.^30^ In addition, PoCUS can increase the diagnostic accuracy for deep vein thrombosis with appropriate provider education training.^29^

### Respiratory

PoCUS is a quick and cost-effective instrument to evaluate patients with respiratory complaints and can be used to assess both acute and chronic respiratory conditions.^1^,^31^ Shortness of breath is a common presenting complaint in primary care; as a result, it can be challenging to distinguish between differential diagnoses using health history and physical assessment alone.^32^ Ultrasound can be used as a means to detect abnormalities within the lungs and quickly and efficiently rule out pneumonia (areas of consolidation) or pleural fluid (effusion). A chest X-ray is the current standard diagnostic imaging ordered to rule out pathologies such as fluid overload, pneumonia, and pleural effusions. However, the accuracy and time to diagnosis of respiratory conditions is far superior with PoCUS when compared with both chest X-rays and auscultation.^25^ In other words, the sensitivity and specificity of PoCUS is significantly higher than that of chest X-rays for both respiratory and certain cardiac processes.^32^

PoCUS can be used by NPs to promptly and accurately identify a variety of respiratory conditions. Ultrasound is beneficial for evaluating pneumonia, given that consolidation typically develops near the surface of the lung.^30^ In addition, Smallwood and Dachsel described the sensitivity of ultrasound for suspected pleural effusions to be three times greater than that of radiography.^30^ With ultrasound training, practitioners can examine for abnormal B lines and selectively assess for or rule out pulmonary edema.^1^,^32^ The increased frequency of B lines noted in patients with pulmonary edema differ and are easily distinguished from the normal A lines seen in healthy patients.^32^ As a result, PoCUS can be used by NPs to selectively identify and differentiate between normal and abnormal respiratory processes.

### Gastrointestinal

NPs utilize ultrasound for various abdominal complaints and gastrointestinal pathologies that extend beyond obstetrics. Unfortunately, patients typically face exceedingly long wait times for outpatient imaging, such as CT scans and ultrasounds, which are the diagnostic gold standard for abdominal complaints. Alternative methods of imaging, such as PoCUS, can rule out processes such as abdominal aortic aneurysm (AAA) in patients without obesity, thus minimizing the impact of wait times for formal diagnostics. In addition, PoCUS for AAA screening has been found to have a sensitivity of 93% and specificity of 97%, which is superior to abdominal palpation alone.^29^,^30^ Given the high mortality associated with AAA, one-time screening of men ages 65 to 80 years is recommended by Canadian preventive health guidelines and of men ages 65 to 75 who have ever smoked by US guidelines.^33^,^34^ Although diagnostic ultrasound is more selective for AAA, PoCUS assessment can allow timely recognition and improve patient adherence to screening and management.^32^,^35^

Generalized abdominal pain is a frequent presenting complaint in primary care. NPs need to be cognizant of red flags that require the pursuit of surgical and emergent interventions. PoCUS has been successfully implemented into primary care to rule out acute abdomen, a condition that often requires timely referral to a higher level of care.^35^ Common emergent conditions associated with abdominal pain include acute appendicitis, acute cholecystitis, renal colic, AAA, and bowel obstruction. A certified PoCUS practitioner can efficiently evaluate for these common urgent pathologies by examining for free air, anatomic abnormalities, hydronephrosis, and other concerning findings.^35^ Certified NPs working in primary care rural settings can utilize PoCUS to evaluate potential red flags and determine necessary transportation costs and patient tribulations.

### Obstetrics and gynecology

Ultrasound, most notably at the bedside, is recognized as a standard diagnostic practice in obstetric and gynecologic care.^29^ Doig et al. emphasized that rural and remote communities are severely underserved with regards to antenatal care.^36^ PoCUS can be used to evaluate and assess for intrauterine pregnancy and rule out red flags such as ectopic pregnancy, fetal complications, or stillbirth.^29^,^35^ Thus, it is an ideal clinical tool for rural and remote communities. PoCUS can decrease some of the healthcare burdens in these communities and improve maternal and fetal patient-care outcomes. In addition, PoCUS can be used in the antenatal period to assess for anomalies as well as the number of fetuses and the approximate gestational age.^35^,^36^ Furthermore, timely ultrasound screening and point-of-care bedside assessment can significantly reduce maternal and fetal risk factors, thus improving morbidity and mortality.^35^-^37^ Primary care NPs can efficiently optimize obstetric assessments using timely bedside ultrasound imaging.

### Musculoskeletal complaints

Bedside ultrasound can increase the diagnostic accuracy for a variety of presenting complaints, including joint and soft-tissue injuries.^29^,^30^ Abscess and cellulitis are two conditions that can be difficult to differentiate in primary care and require different treatment strategies (that is, incision/drainage and antibiotics, respectively).^38^ PoCUS would allow the NP to accurately diagnose an abscess and identify the need for incision and drainage, effectively avoiding unnecessary or potentially futile treatments.^38^ Joint effusions can be assessed using bedside ultrasound; PoCUS can be used to assist in aspirating joint fluid for further lab evaluation by visualizing the targeted area.^30^ The use of ultrasound may also allow visualized guidance for corticosteroid joint injections. PoCUS promotes accurate diagnosis of musculoskeletal complaints, thereby reducing the incidence of misdiagnosis.

## Education, training, and safety

The clinical accuracy of PoCUS depends on the qualifications, training, and certification of the provider. Providers must acknowledge their limitations and participate in ongoing professional development to ensure accurate application, interpretation, and competency.^3^,^13^,^26^,^39^ The use of PoCUS requires a unique skill set that includes proficiency, clinical assessment, and interpretation. Misinterpretation and misdiagnoses can pose significant risk to the patient.^39^ The accuracy of PoCUS is associated with the anatomic area and the extent of the exam, and is directly related to the individual education and interpretation that is reflected in a certified practitioner's familiarity and confidence.^26^,^39^

## Conclusion

The implementation of PoCUS in NP primary care practice is associated with improved patient outcomes, enhanced treatment accuracy, reduced treatment failure rates, lower healthcare expenditures, and improved time to diagnosis. PoCUS can complement clinical assessments in various healthcare sectors and profoundly benefit communities with limited resources and access to formal diagnostic imaging. PoCUS is a promising emerging solution that can decrease health inequities in rural and remote communities. Research has confirmed that PoCUS is a useful, cost-effective, and precise diagnostic tool when used by educated and certified users. Beyond a need for further research of its benefits and limitations for practice, PoCUS education offers a unique opportunity to develop and research interprofessional health collaborations. NPs employed in areas such as ambulatory or primary care would benefit from implementing PoCUS into their practice to concurrently improve patient satisfaction and strengthen therapeutic relationships.

## PoCUS benefits and limitations in primary care^1^–^3^,^7^,^9^,^12^,^19^,^20^,^26^

**Benefits**

- Convenient access and portability
- Cost-effective
- Decreased wait times compared with conventional diagnostic imaging
- Expeditious evaluation for potential red flags
- Interprofessional use and collaboration (RN, NP, physician, radiology technician)
- No exposure to harmful radiation as with certain other formal diagnostic testing (such as CTs)
- Reduction in unnecessary transportation and relocation burden for rural or remote populations
- Timely diagnosis without compromising clinical accuracy

**Limitations**

- Education is essential to ensure clinical application and interpretation accuracy
- Education and interpretation can vary in complexity; therefore, it is necessary for anatomic-specific training to be individualized in order to ensure accuracy
- PoCUS procedure education may be cost-prohibitive
- PoCUS is not to be used as a replacement for radiologist-interpreted images when they are indicated
- Misinterpretation of PoCUS results can occur if not limited to the clinical anatomic context of concern

Abbreviations: CT, computed tomography; PoCUS, point-of-care ultrasound.

## Indications for use of ultrasound in primary care^1^,^4^,^28^,^31^

- Cardiovascular
  – Pericardial effusion
  – Decreased systolic function
  – Heart failure
  – Deep vein thrombosis
- Respiratory
  – Areas of consolidation
  – Pleural effusion
  – Pulmonary edema
- Gastrointestinal
  – Abdominal aortic aneurysm
  – Bowel obstruction
  – Acute appendicitis
  – Acute cholecystitis
  – Renal colic
- Obstetrics and gynecology
  – Intrauterine versus ectopic pregnancy
  – Anatomic complications
  – Fetal heart activity
  – Approximate gestational age
  – Number of fetuses
- Musculoskeletal
  – Soft-tissue injuries
  – Joint injuries
  – Joint effusion
  – Cellulitis versus abscess
